# Supplementary figures and images for: Balancing the supply and demand for taxonomy: An analysis of European taxonomic capacity and policy needs
Source: PLoS One. 2026 Apr 20;21(4):e0347332. doi: 10.1371/journal.pone.0347332 (PMC13095113; doi:10.1371/journal.pone.0347332)

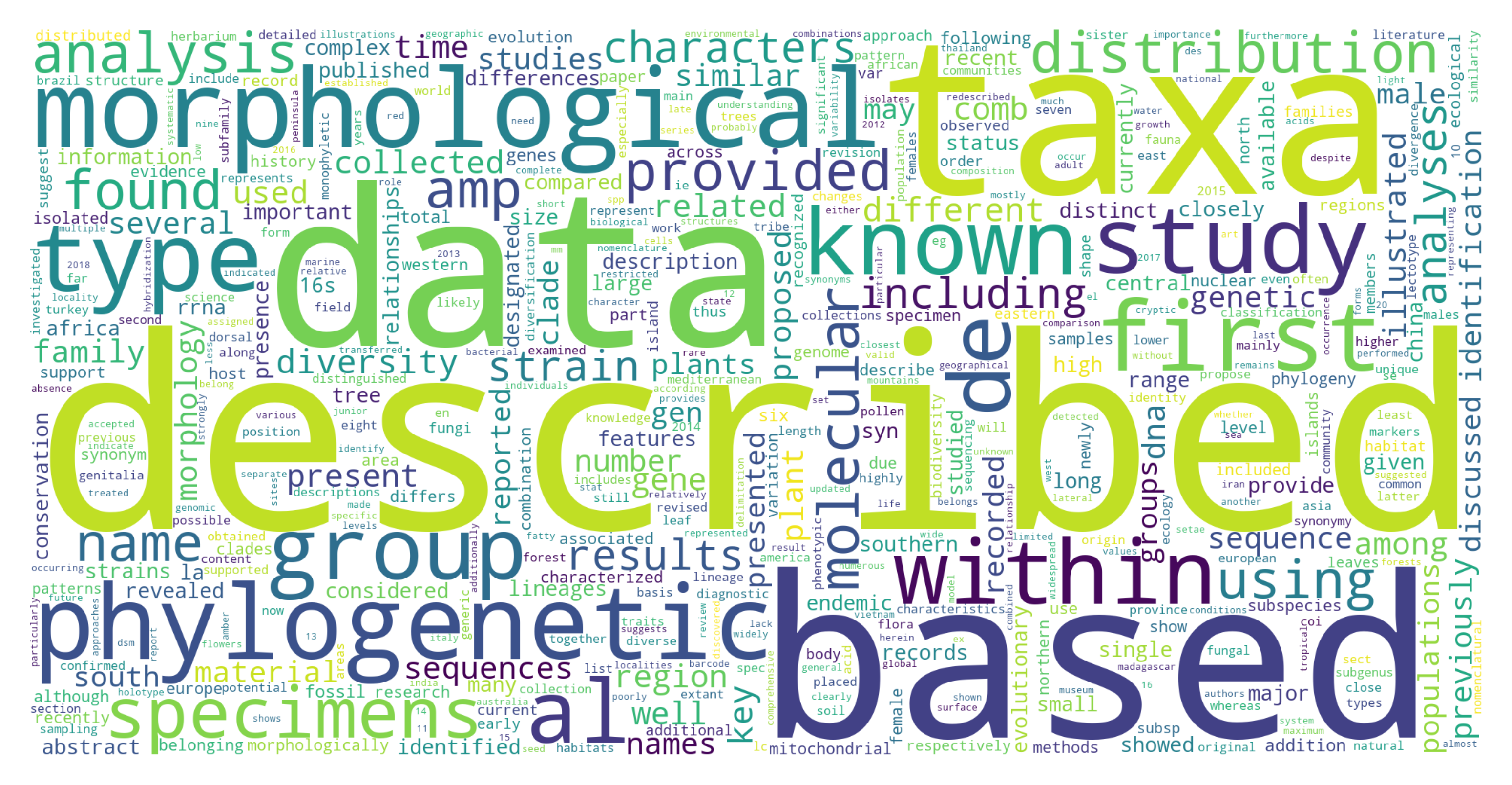

Supplement: S1 Fig — This gives a visual representation of the subjects of the articles and a qualitative check that we have predominantly filtered for taxonomic articles. (TIF) [file pone.0347332.s001.tif]

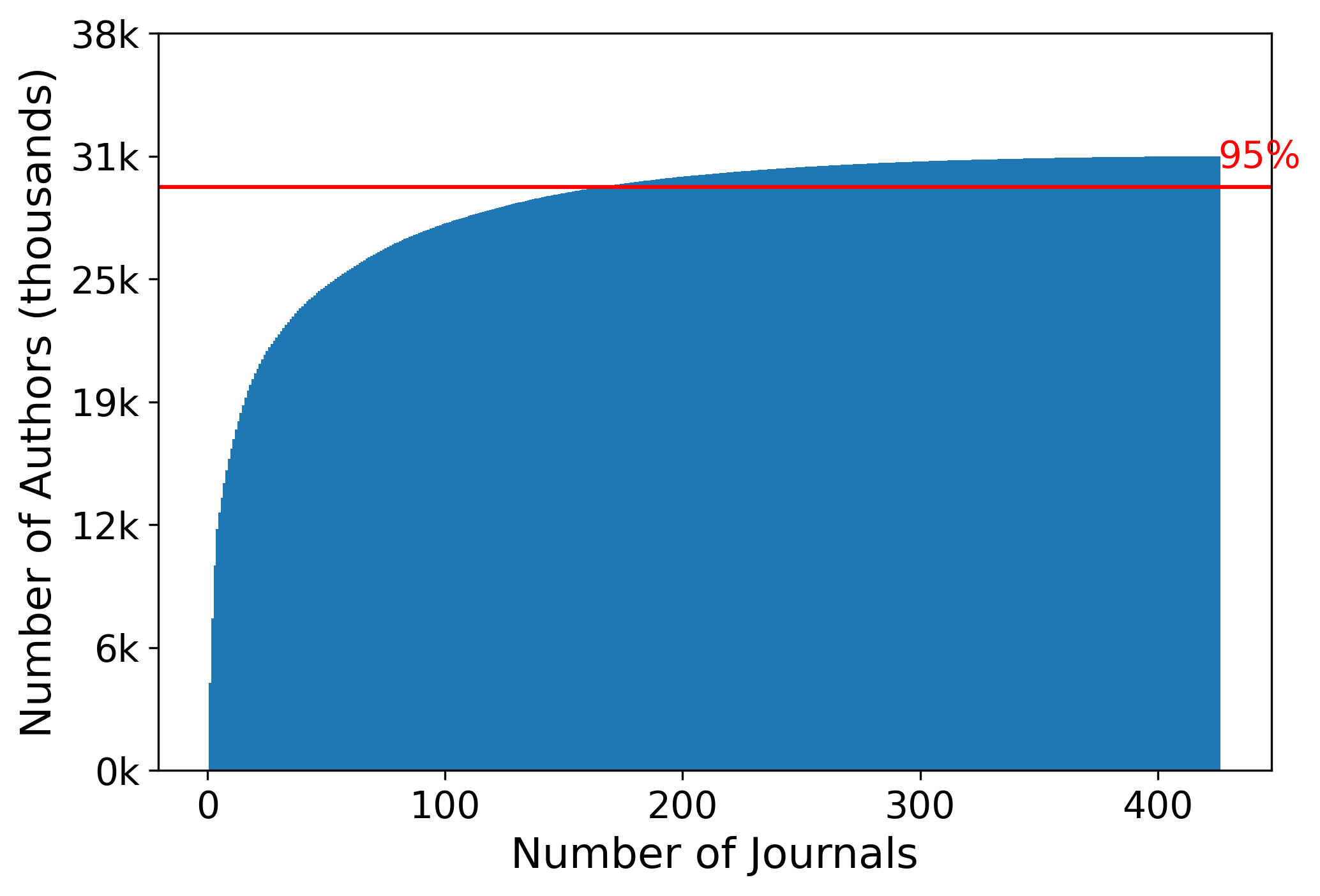

Supplement: S2 Fig — (TIF) [file pone.0347332.s002.tif]

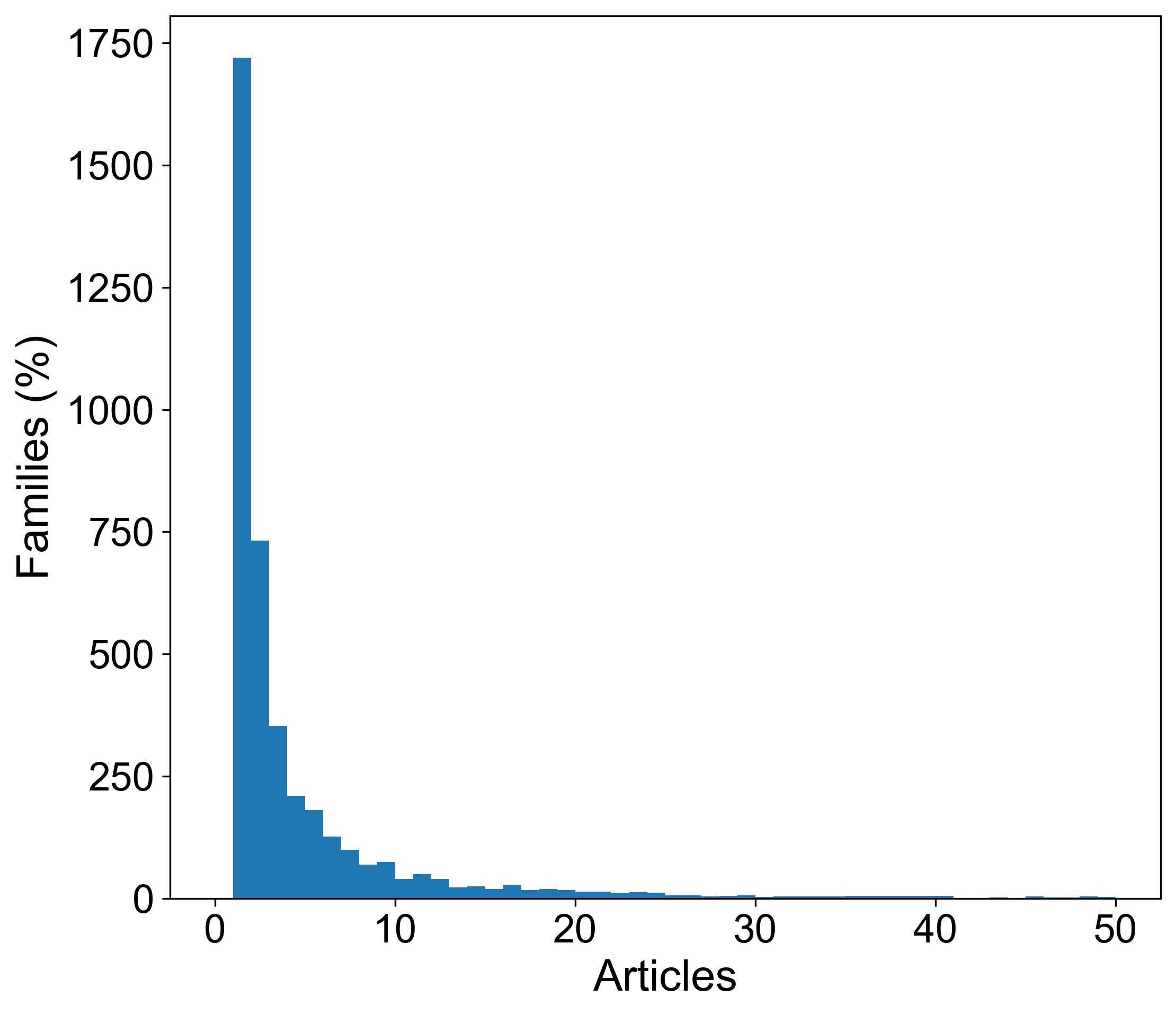

Supplement: S3 Fig — The top 10 families by number of articles are Asteraceae: 400, Staphylinidae: 311, Fabaceae: 297, Orchidaceae: 297, Poaceae: 262, Scarabaeidae: 173, Curculionidae: 168, Lamiaceae: 150, Erebidae: 145, Caryophyllaceae: 139. (TIF) [file pone.0347332.s003.tif]

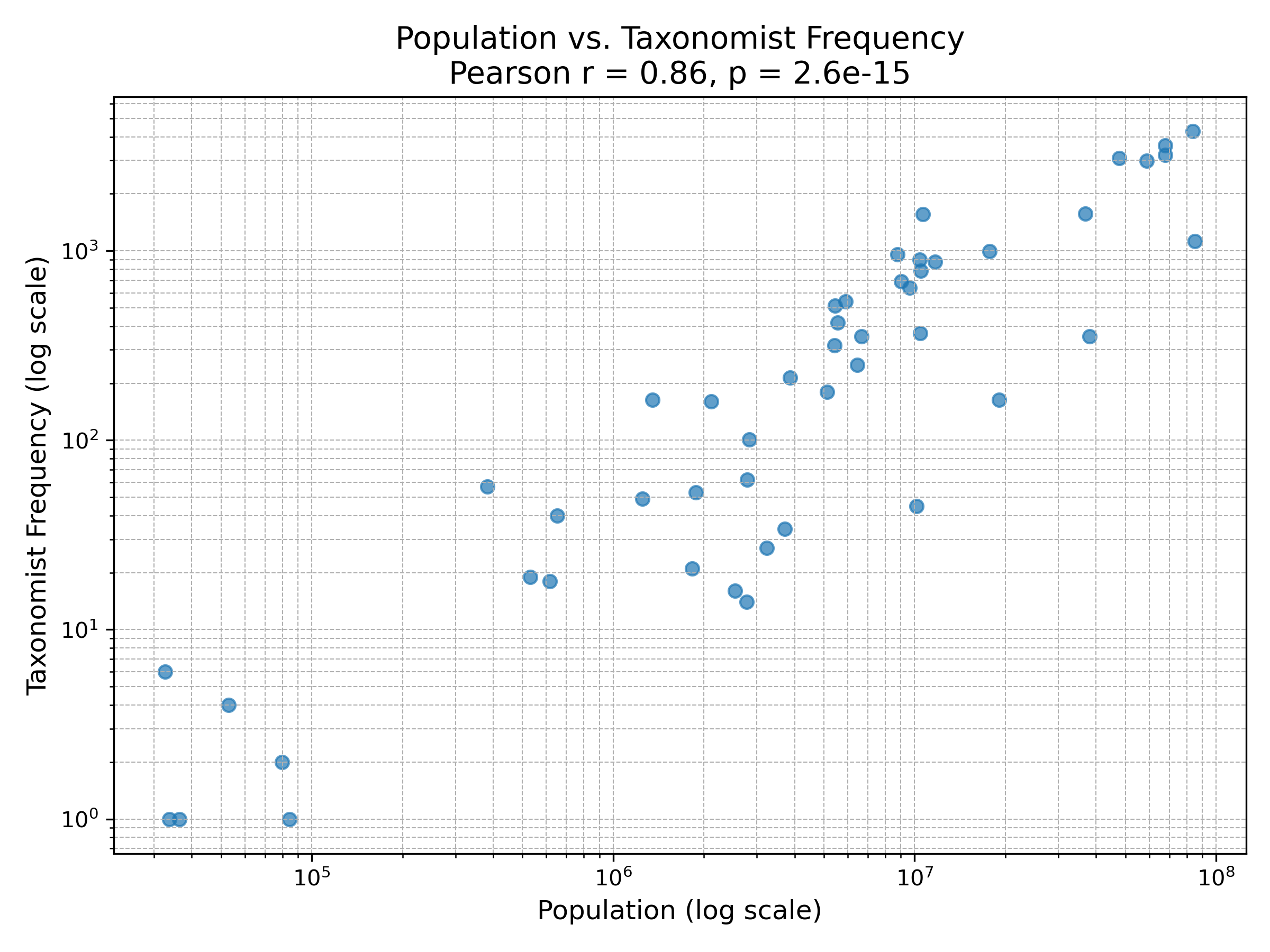

Supplement: S4 Fig — Pearson correlation coefficient: 0.8640 (p = 2.634e-15). (TIF) [file pone.0347332.s004.tif]

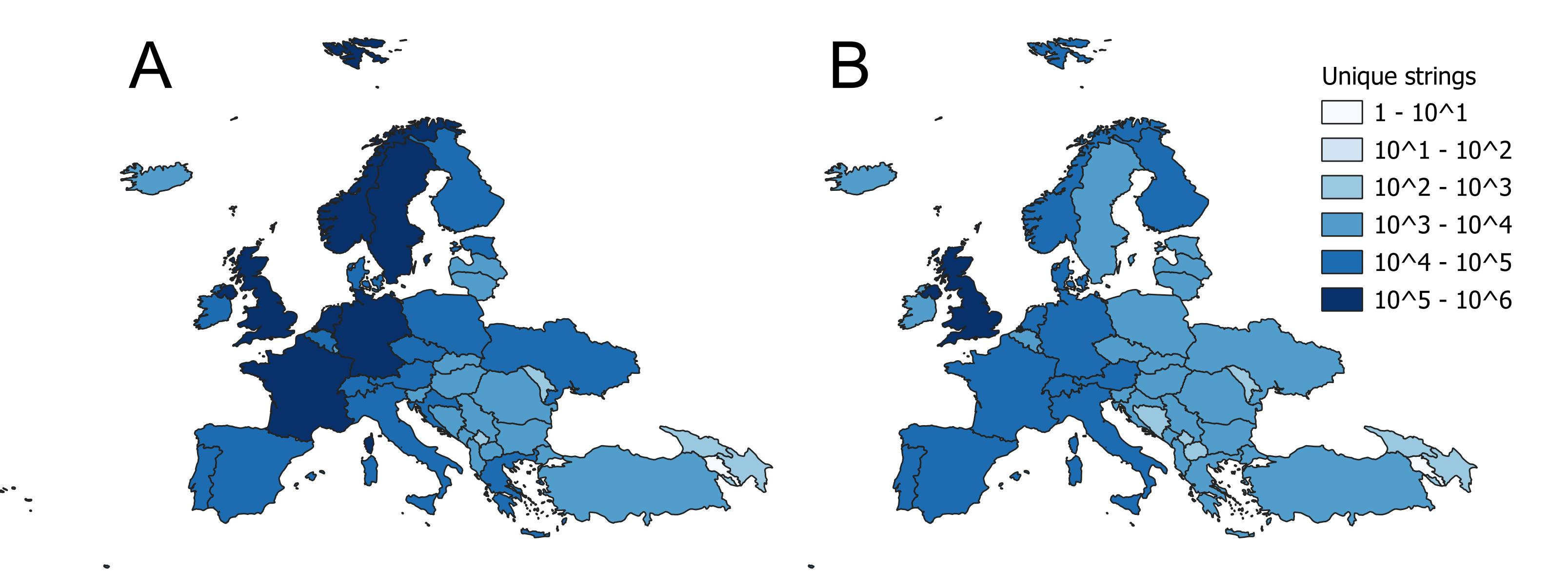

Supplement: S5 Fig — (A) and dwc:identifiedBy text strings (B) on occurrences from GBIF per country from years between 2014 and 2023 inclusive. Note that the colour ramp is on a logarithmic scale and that the maps use an equal area Mollweide projection. Made with Natural Earth. Free vector and raster map data @ naturalearthdata.com. (TIF) [file pone.0347332.s005.tif]

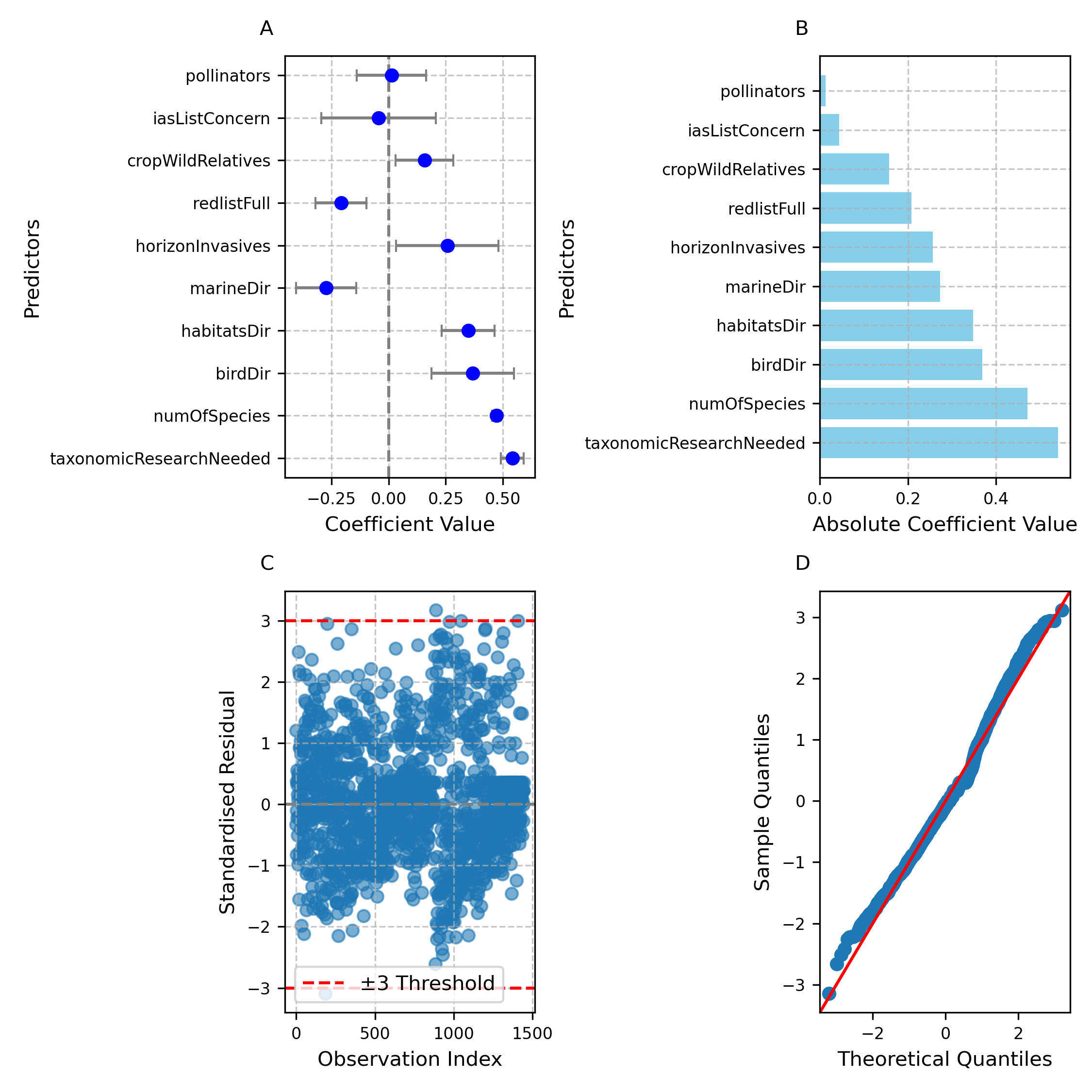

Supplement: S6 Fig — (A) Estimated regression coefficients (±95% confidence intervals) for the model relating taxonomic research effort (number of authors) to species richness and policy variables. Predictors are ordered by absolute importance. (B) Relative importance of predictors based on the absolute value of their standardised regression coefficients. (C) Standardised residuals from the robust regression model, with horizontal red lines indicating thresholds for potential outliers (residual > 3). (D) Q–Q plot showing the quantiles of the residuals against the theoretical quantiles of a normal distribution. Deviation from the 45° reference line indicates departures from normality. (TIF) [file pone.0347332.s006.tif]

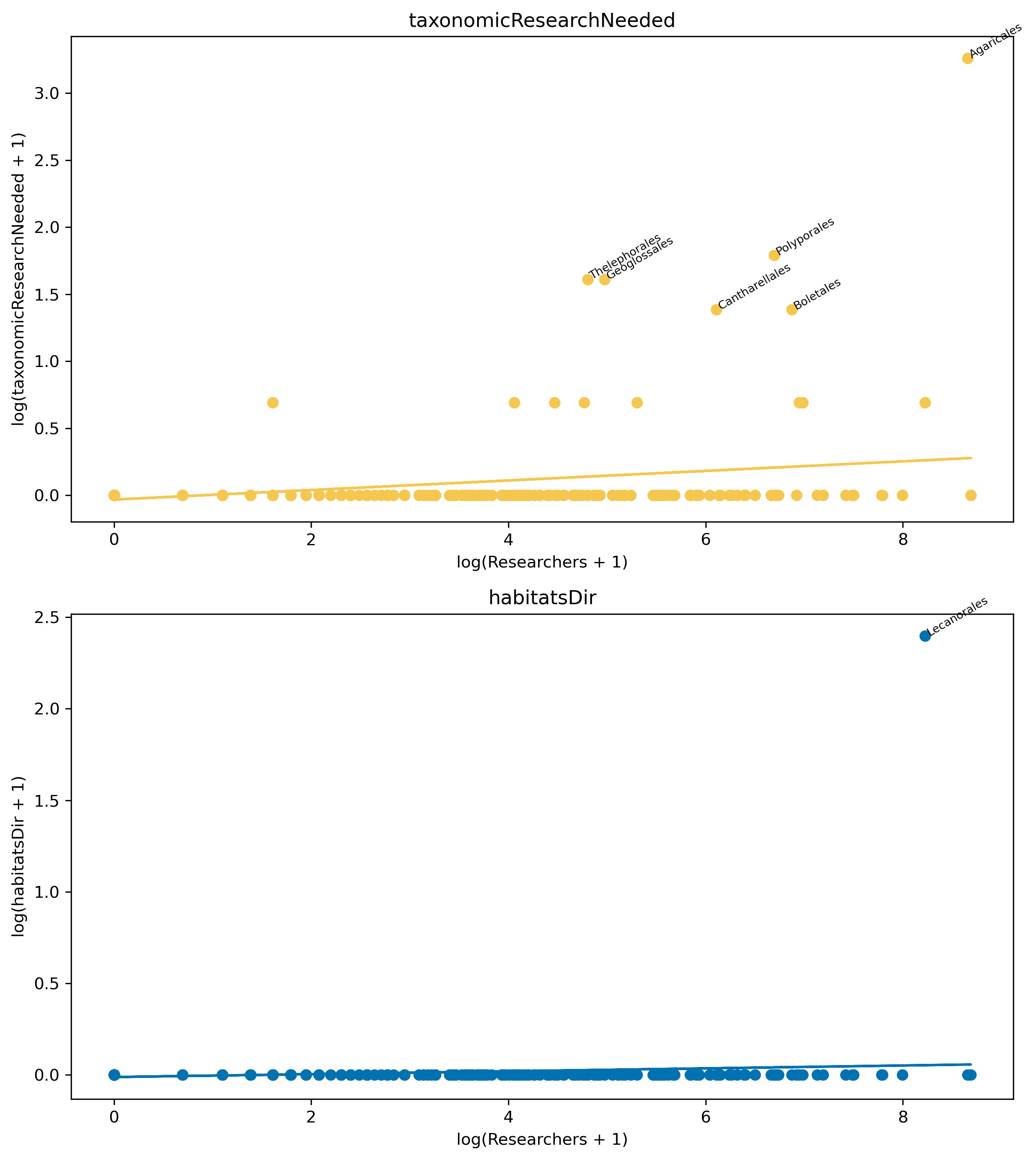

Supplement: S9 Fig — Each plot corresponds to a different policy included in this study. (TIF) [file pone.0347332.s009.tif]
