# Supplementary material for: Balancing the supply and demand for taxonomy: An analysis of European taxonomic capacity and policy needs
Source: PLoS One. 2026 Apr 20;21(4):e0347332. doi: 10.1371/journal.pone.0347332 (PMC13095113; doi:10.1371/journal.pone.0347332)
Supplement: S1 Table — Numbers in parentheses indicate the number of species in each taxon. (PDF) [file pone.0347332.s010.pdf]

Table S1: The most common taxa in the European red list, the European red list taxonomic research needed category, the list of European crop wild relatives and invasive species on a horizon scanning list. The number of each species in each taxa are in parentheses.

Rank	Red List - top ten families	Taxonomic research needed - top ten families	Crop wild relatives - top ten genera	Invasive species on the horizon - top nine families
1	Hydrobiidae (588)	Hydrobiidae (80)	Trifolium (71)	Formicidae (5)
2	Apidae (561)	Hydnaceae (71)	Medicago (24)	Cichlidae (4)
3	Andrenidae (465)	Moitessieriidae (55)	Vicia (21)	Poaceae (4)
4	Megachilidae (442)	Geomitridae (54)	Brassica (19)	Sciuridae (4)
5	Hygromiidae (397)	Tettigoniidae (42)	Avena (18)	Ampullariidae (3)
6	Tettigoniidae (350)	Hygromiidae (41)	Lathyrus (15)	Asteraceae (3)
7	Acrididae (334)	Amaryllidaceae (41)	Lotus (15)	Celastraceae (3)
8	Halictidae (314)	Rosaceae (40)	Prunus (15)	Cerambycidae (3)
9	Cerambycidae (278)	Clausiliidae (39)	Aegilops (14)	Fabaceae (3)
10	Cyprinidae (238)	Helicidae (37)	Allium (14)	–
